# Accurate and transferable drug–target interaction prediction with DrugLAMP

**DOI:** 10.1093/bioinformatics/btae693

**Published:** 2024-11-21

**Authors:** Zhengchao Luo, Wei Wu, Qichen Sun, Jinzhuo Wang

**Affiliations:** Department of Big Data and Biomedical AI, College of Future Technology, Peking University, Beijing 100871, China; Department of Big Data and Biomedical AI, College of Future Technology, Peking University, Beijing 100871, China; School of Mathematical Sciences, Peking University, Beijing 100871, China; Department of Big Data and Biomedical AI, College of Future Technology, Peking University, Beijing 100871, China

## Abstract

**Motivation:**

Accurate prediction of drug–target interactions (DTIs), especially for novel targets or drugs, is crucial for accelerating drug discovery. Recent advances in pretrained language models (PLMs) and multi-modal learning present new opportunities to enhance DTI prediction by leveraging vast unlabeled molecular data and integrating complementary information from multiple modalities.

**Results:**

We introduce DrugLAMP (PLM-assisted multi-modal prediction), a PLM-based multi-modal framework for accurate and transferable DTI prediction. DrugLAMP integrates molecular graph and protein sequence features extracted by PLMs and traditional feature extractors. We introduce two novel multi-modal fusion modules: (i) pocket-guided co-attention (PGCA), which uses protein pocket information to guide the attention mechanism on drug features, and (ii) paired multi-modal attention (PMMA), which enables effective cross-modal interactions between drug and protein features. These modules work together to enhance the model’s ability to capture complex drug–protein interactions. Moreover, the contrastive compound-protein pre-training (2C2P) module enhances the model’s generalization to real-world scenarios by aligning features across modalities and conditions. Comprehensive experiments demonstrate DrugLAMP’s state-of-the-art performance on both standard benchmarks and challenging settings simulating real-world drug discovery, where test drugs/targets are unseen during training. Visualizations of attention maps and application to predict cryptic pockets and drug side effects further showcase DrugLAMP’s strong interpretability and generalizability. Ablation studies confirm the contributions of the proposed modules.

**Availability and implementation:**

Source code and datasets are freely available at https://github.com/Lzcstan/DrugLAMP. All data originate from public sources.

## 1 Introduction

The task of discovering a new drug necessitates a blend of research expertise, intelligence, courage, and serendipity. Over 665 000 drug compounds, containing more than 5000 potential target proteins, have been confirmed by the United States Food and Drug Administration (Isert *et al.* 2022, Svilenov *et al.* 2023), yet fewer than 15 first-in-class drugs get approval from the United States Food and Drug Administration annually (Mullard 2018). Traditional in vivo drug testing, which is both costly and time-consuming (Dickson and Gagnon 2004, Lee *et al.* 2019), presents challenges to large-scale exploration of novel drugs (Vincent *et al.* 2022). Moreover, the patients involved in these tests face considerable toxicity risks due to the use of immature and unfamiliar drugs (Rostami-Hodjegan and Tucker 2007). As an alternative, computational approaches narrow down the number of compounds to be considered and offer insights into drug–target interactions. Although high-throughput screening through in vitro assays is commonly employed, it is an expensive and limited method to comprehensively screen massive drug databases. Identifying new DTIs remains a vital stage in drug discovery (Zheng *et al.* 2020). Despite the substantial associated costs and time, laboratory-based compound–protein interaction measurement remains the gold standard (Lee *et al.* 2019). The use of computational methods to predict DTIs, however, can accelerate the drug discovery process and cut down on capital investment (Schneider 2018).

DTI prediction has been a focal point in computational drug discovery for many years. Traditional machine learning-based methods typically rely on hand-crafted features and classical algorithms. These include similarity-based methods, which utilize chemical structure similarity of drugs and sequence similarity of targets to predict interactions, such as the supervised bipartite graph inference method (Yamanishi *et al.* 2008) based on chemical and genomic data. Another approach involves kernel-based methods, which leverage various kernels to capture drug and target similarities, exemplified by Gönen’s kernelized Bayesian matrix factorization method (Gönen 2012) for DTI prediction. However, these traditional methods often struggle with the high-dimensional nature of drug and protein data and may not capture complex, non-linear relationships effectively.

With advancements in deep learning technology, numerous attempts have been made to effectively employ these techniques for DTI problems. Deep learning methods can automatically learn features of drugs and proteins from DTI data, rather than relying solely on predefined descriptors (Cortes and Vapnik 1995, Ho 1995). Due to the limited availability of biological targets with accessible 3D structures, many deep learning methods use linear or two-dimensional structural information of drugs and proteins as input.

These deep learning approaches leverage neural networks to automatically learn features from raw data. Convolutional neural networks (CNNs) have been widely adopted, with examples like DeepDTA (Bagherian *et al.* 2021) using CNNs to process both drug and protein sequences for DTI prediction. Similarly, DeepConv-DTI (Lee *et al.* 2019) employs CNNs to process protein sequences and molecular fingerprints for drug–target interaction prediction.

Graph neural networks (GNNs) have also shown promise in this field. GraphDTA (Nguyen *et al.* 2021) uses GNNs to capture the structural information of drugs represented as molecular graphs. Taking this approach further, MGraphDTA (Yang *et al.* 2022) introduced a deep multi-scale graph neural network for explainable drug–target binding affinity prediction, incorporating 3D protein structure information.

Attention-based models have gained significant traction in recent years (Xia and Wang^2023^). Shin introduced a self-attention-based molecule representation for predicting drug–target interaction (Shin *et al.* 2019), which can capture long-range dependencies in molecular structures. TransformerCPI (Chen *et al.* 2020) leverages a transformer architecture with self-attention mechanism and employs label reversal experiments to improve compound–protein interaction prediction. MolTrans (Huang *et al.* 2021) incorporates attention mechanisms to focus on important substructures in drugs and proteins, further advancing the field.

While these deep learning methods show improved performance, they are not without limitations. They often require large amounts of labeled data and may not generalize well to novel drugs or targets. Nevertheless, these approaches represent significant progress in the field of DTI prediction and continue to push the boundaries of what’s possible in computational drug discovery.

PLMs have found success in fields related to natural language processing (Devlin *et al.* 2018, Radford *et al.* 2018, Yang *et al.* 2019). PLMs leverage large volumes of data and are trained in a self-supervised manner (Wei *et al.* 2022). The data used do not require expensive or professional labeling. PLMs are recognized for their impressive encoding ability and have demonstrated excellent generalization performance across a multitude of downstream tasks (Liu *et al.* 2019). Recently, large-scale pretraining of graph models has gained popularity (Hou *et al.* 2022). Given the similarities between 2D structures of molecules and graphs, sequences of proteins and languages, we propose that PLMs need to extend to the domain of binding problems involving molecules and proteins. There have been studies utilizing PLMs in DTI prediction (Kang *et al.* 2022, Lee *et al.* 2024). However, the application of PLMs to construct multiple modalities for DTI prediction remains unexplored.

Multi-modal fusion technology, advanced technology underpinned by attention mechanisms (Vaswani *et al.* 2017), forms the architectural backbone of existing PLMs. Using attention mechanisms, data in multiple modalities—such as images, text, and sounds—can be efficiently processed (Radford *et al.* 2021, Guzhov *et al.* 2022, Girdhar *et al.* 2023). By paying attention and focusing on information from different modalities, the technology can extract key features, resulting in more accurate and comprehensive analytics and reasoning. Additionally, multi-modal fusion technology is uniquely suited to address the inherent characteristics of DTI challenges. The DTI problem refers to the problem of predicting the interaction between drugs and proteins (Zheng *et al.* 2020), and multi-modal fusion technology can predict the interaction between them by combining data from different modalities, such as the structural information of the drug and the sequence information of the protein. Only methods that integrate multiple data types can enhance the accuracy and reliability of predictions.

Contrastive learning boasts several advantages in the machine learning field, and it is widely employed in PLM training (Radford *et al.* 2021). This technology allows for the training and optimization of complex models using unsupervised learning methods, thus reducing data collection and labeling costs compared to traditional supervised learning methods. Furthermore, contrastive learning can align and constrain multiple modalities (Li *et al.* 2021), allowing correlations and shared representations between different modalities to be learned, which aids the model in better understanding and processing multi-modal data. This capability is a critical element in DTI problems that require input multi-modal information.

Some methods utilizing PLMs have emerged to capture rich molecular and protein representations for drug–target interaction prediction. Kang *et al.* (2022) fine-tune BERT-like models for both drugs and proteins, leveraging BERT’s ability to process sequential data and encode drug SMILES strings and protein sequences. This approach aims to capture complex patterns in both molecular and protein data. Lee *et al.* (2024) introduce DLM-DTI, a dual language model with hint-based learning. This model uses two interconnected language models to simultaneously process drug and protein information, allowing for modality-specific feature extraction and cross-modal interaction learning. While these PLM-based methods show promise, they often treat drug and protein modalities separately and may not fully capture their complex interactions. Additionally, their performance can depend heavily on pretraining data quality and diversity.

As we delve deeper into the practical applications and broader implications of above methods, several critical challenges remain:

1. Data scarcity in the DTI field: Construction of DTI datasets requires labels from real drug and target experiments. This process is usually time-consuming and laborious (Paul *et al.* 2010). The significant financial and time-costs make drug discovery a notoriously high-risk field. For methods based on neural networks, insufficient data also limits the enhancement of accuracy and generalization capabilities for DTI problems.
2. Inadequate generalization capabilities in existing methods: Often, researchers face newly discovered protein targets or newly developed drugs in drug discovery (Hughes *et al.* 2011, Pahikkala *et al.* 2015). Existing methods cannot be applied to actual drug discovery scenarios because of insufficient generalization.
3. Limited exploration of DTI multi-modal fusion: Existing methods generally consider only a single modality (Öztürk *et al.* 2018, Öztürk *et al.* 2019) but the actual interaction between small molecules and drugs encompasses a complex mix of topology, patterns, and 3D physical information (Cheng *et al.* 2007). Single modality input results in incomplete information. While single-modality, data-based DTI prediction is possible, furthering the improvement of accuracy requires the integration of complementary multi-modal information.

With the rapid advancements in PLM, multi-modal fusion, and contrastive learning, these DTI challenges can be overcome. This study investigates the application of large-scale molecule and protein models to multi-modal DTI predictions, and assesses the performance of such a model in real-world situations. Through the utilization of a 2C2P module, which effectively compares different modalities at multi-scale levels, we evaluate the model’s performance in two real-world drug discovery scenarios.

Our study demonstrably extends the encoding ability of PLMs beyond the scenario of standard DTI computational performance testing metrics. We provide a practical scenario to demonstrate its adaptability to real-world drug discovery scenarios, showcasing its ability to learn from vast multi-modal drug–target data. This broad applicability underlines the potential of our predictive model to facilitate novel drug discovery, consequently opening new possibilities for optimization and innovation. We contribute the following key findings to this field:

1. Our research explores multiple modalities for DTI prediction. By integrating various PLMs, we enhance the accuracy and reliability of our predictive models, significantly furthering DTI prediction.
2. Introduction of multi-modal fusion modules, such as the PMMA and PGCA, considerably improves the model’s generalization performance. Particularly, the utilization of the 2C2P module enables application of our model in real-world scenarios.
3. Our research extends beyond model predictive performance and demonstrates the interpretability and potential of the model. We used the model to conduct adverse event inference and cryptic pockets prediction, and checked visualization to reveal the intrinsic mechanism of the model in predicting the interaction between drugs and proteins, which is crucial to the broader understanding of DTI-related issues.
4. To foster academic exchange and research advancement, our model has been made open source. This release affords other researchers the freedom to engage with the model in their studies and experiments, fostering collective growth of the field. Researchers can introduce cutting-edge PLM advances into the DrugLAMP framework and modify some components to achieve never-ending learning (Mitchell *et al.* 2018).

## 2 Materials and methods

### 2.1 Datasets

To evaluate DrugLAMP’s performance, we used three data split settings: random split, cold-start split, and cluster-start split. The latter two are designed to simulate real-world drug discovery scenarios, progressively increasing the difficulty of the DTI prediction task.

The random split is the conventional setting where data are randomly divided into training, validation, and test sets. While useful for initial evaluation, it does not reflect the challenges of predicting interactions for novel drugs or targets.

The cold-start setting simulates predicting interactions for completely new drugs or targets unseen during training. This is crucial in real-world drug discovery, where we often encounter novel compounds or newly discovered protein targets. In this setting, we set aside a portion of drugs and targets for validation and testing, using the remainder to construct the training set.

The cluster-start setting takes this concept further by ensuring that training and test data come from different regions of the chemical and protein space. We first cluster drugs and targets separately based on structural or sequence similarities, then split these clusters into training and testing groups. This evaluates the model’s ability to generalize to structurally distinct drugs and targets. Detailed procedures for data preprocessing and split generation can be found in the Supplementary Materials, “Dataset Settings” Section.

We selected the BindingDB, BioSNAP, Human and Kinase datasets to assess the performance of our model. The BindingDB is a publicly accessible database that focuses on proteins that are drug–targets or candidate drug–targets. We used a version constructed by Bai *et al.* (2021). The BioSNAP dataset consists of 27 464 drug–target pairs for 4505 drugs and 2181 proteins. The Human dataset is constructed based on highly credible negative DTI samples and includes 6728 interactions between 2726 drugs and 2001 proteins. The Kinase dataset is constructed by Chen *et al.* (2020) based on the KIBA (Tang *et al.* 2014) dataset and includes 1644 compounds plus 229 proteins. The statistical details of the datasets can be found in Supplementary Materials, Tables S2 and S3.

### 2.2 Problem formulation

In DTI prediction, we consider the task as a binary classification problem, where the goal variables are all in {0,1}. The single input to the problem is a drug-target pair, where the drug is represented as $S^{D}$ by the SMILES and the target is denoted as $S^{P}$ by the primary sequence of the protein. Each token in the protein sequence represents one of the 23 amino acids. SMILES is a specialized sequence format obtained through a depth-first search of the two-dimensional molecular graph, which includes tokens for chemical atoms and bonds (e.g. C, N, O). However, since computers are not sensitive to the two-dimensional implications contained in one-dimensional sequences, we convert SMILES back to 2D molecular graphs G={V,E}, where V represents vertices (atoms) and E represents edges (chemical bonds).

To obtain more reliable features, we utilized extractors and PLMs to extract features from drug and protein separately. Our goal is to obtain the mixed drug-protein features by extractors and PLMs and simulate their interactive behavior, and finally predict whether the interaction between the drug–target pair can occur.

Given the SMILES sequence ${\text{S}}^{D}=\{S_{1}^{D},S_{2}^{D},\ldots ,S_{M}^{D}\}$ of M drug compounds, and the amino acid sequence ${\text{S}}^{P}=\{S_{1}^{P},S_{2}^{P},\ldots ,S_{N}^{P}\}$ of N protein targets, the DTI prediction task can be transformed into learning a projection f that includes the biochemical and structural features of the drug and the protein, and the interaction between the two: $f:{\text{S}}^{D}\times {\text{S}}^{P}\Rightarrow {\mathbb{R}}^{\left(M\times N\right)\times h}$, where h is the dimension of the final mixed vector that combines multi-modal information. These M×N vectors can eventually be passed through a simple multi-layer nonlinear perceptron to get the interaction probability scores of the drug compounds against the target proteins $\text{p}\in {\left[0,1\right]}^{M\times N}$. Finally, we optimize our model’s learnable parameters through back-propagation. The training objective is to minimize the following binary cross-entropy loss: $L_{\mathrm{BCE}}\left(\text{p},Y\right)=-\sum_{p,y\in \text{p},Y}\left(\mathrm{ylog}\left(p\right)+\left(1-y\right)\;\text{log}\;\left(1-p\right)\right)$, where Y is the goal variables. Notations commonly used in this paper are provided in Supplementary Materials, Table S1.

### 2.3 DrugLAMP framework overview

**Figure 1.**
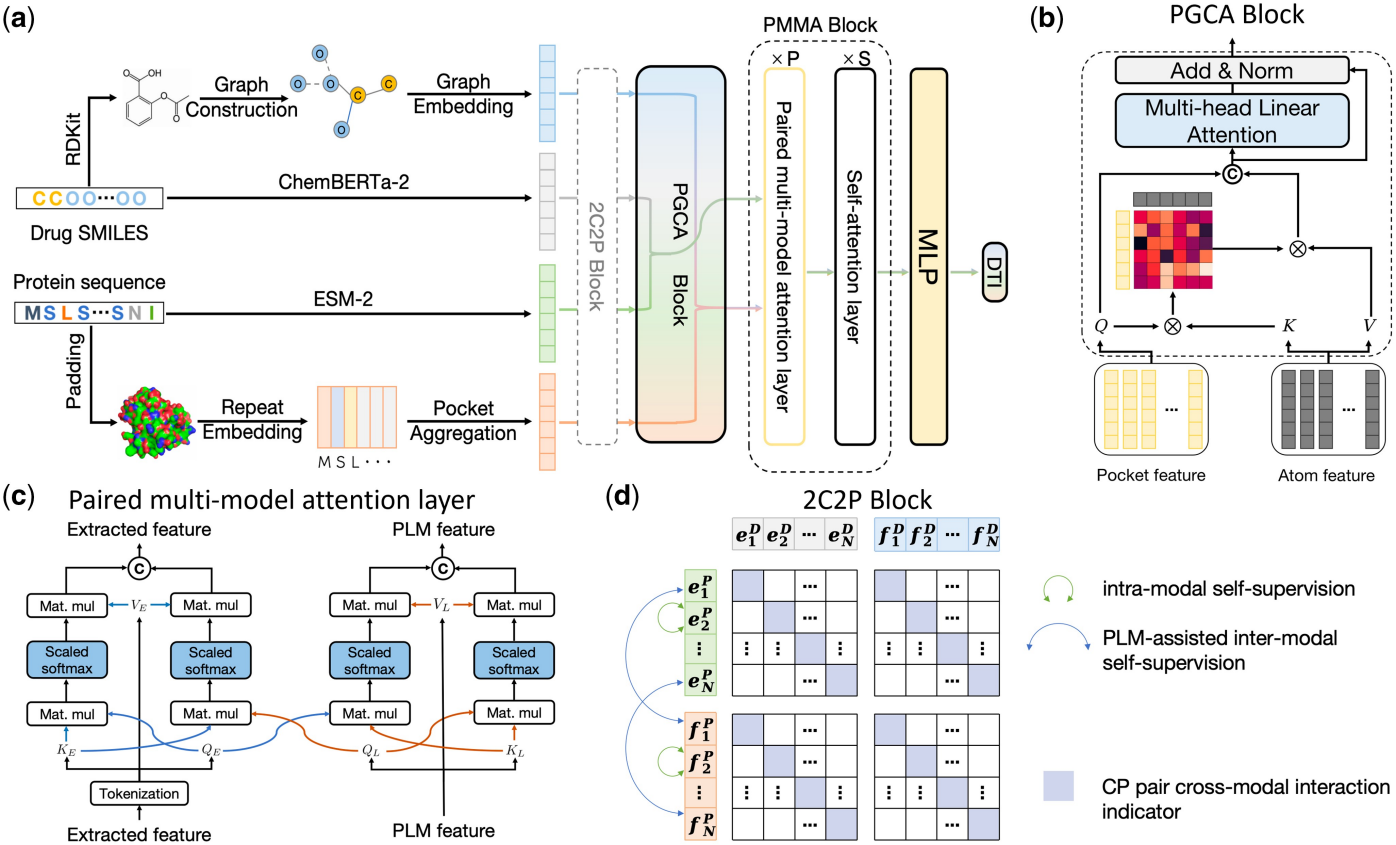
The overarching framework of DrugLAMP. (a) Basic process of DrugLAMP, including multiple modalities construction, multi-modal fusion, and final DTI prediction. The 2C2P module is added to enhance the model's generalization ability in real-world scenarios. (b) Details of the PGCA module, which uses pocket features as queries and atom features as keys and values to guide atom features, concatenating them with pocket features to effectively fuse drug and target features via skip connections and multi-head attention. (c) Details of the PMMA layer, which fuses features extracted by two methods with reservations in a complementary manner. Features from the PLM and feature extractor serve as queries for themselves and each other, then concatenated after scaled dot-product attention (Vaswani *et al.* 2017). (d) Details of the 2C2P module, comparing drug–target information at multiple scales. Intra-modal learning with protein masked language modeling (MLM) at the micro level, inter-modal learning assisted by PLM for drug cross-encoding at the meso level, and cross-modal triplet loss at the macro level.

Taking a drug–target pair as input, the overall framework of DrugLAMP is demonstrated in Fig. 1a. Initially, we utilize graph convolutional network (GCN), 1D convolutional neural networks, and two PLMs to encode the input molecular graph and the target protein sequence. Then, using the encoded protein pocket embeddings, we screen the extracted molecular features output via the co-attention mechanism PGCA in Fig. 1b. Following this, DrugLAMP performed multi-modal fusion through the PMMA module depicted in Fig. 1c. The PMMA module outputs a joint feature that incorporates multi-modal interactions with their respective features, which are then converted into predicted drug–target interaction scores by the subsequent multi-layer perceptron. To amplify DrugLAMP’s robustness in accurately predicting DTI in real-world drug discovery scenarios, we introduced an extra component known as the 2C2P module (Fig. 1d). This module integrates self-supervised learning techniques for drugs and proteins, in addition to cross-modal contrastive learning. The former ensures greater consistency in the feature space between different encoding methods (feature extractors and PLMs), thereby strengthening the model’s generalization ability via label-free pre-training. The latter categorizes the relationship between drugs and proteins into anchor, positive, and negative samples, effectively separating non-interacting sub-samples while maximizing the aggregation of positive samples with known interactions.

### 2.4 PLMs for target and drug

#### 2.4.1 PLM for target

ESM-2 (Lin *et al.* 2022) is a general-purpose protein language model trained on protein sequences from the UniRef (Suzek *et al.* 2015) database and has similar accuracy to AlphaFold2 (Jumper *et al.* 2021) and RoseTTAFold for sequences with low perplexity that are well understood by the language model. The overall distribution of the output feature space of the pretrained model is more in line with the training data set, while maintaining the general representation ability learned in massive data, so that ESM-2 can be better at zero-shot embedding. According to recent benchmarking studies (Lin *et al.* 2023), we choose ESM-2 to obtain the pretrained protein model embeddings $M^{P}$ of its last layer, which can effectively learn information of the three-dimensional structure of a protein.

For the input sequence of ESM-2, we repeatedly padded the input sequence inspired by the following biological facts: drugs may only bind to a specific site on the protein that account for about 10 to 20 percentages and exert their effects (Bugg 2012). And using repeat sequence can enhance the potential for computing each protein site sequence with drug sequence to simulate each possible binding mode, leading to better matching results. We first add front and back gap zeros to the original protein sequence. Then, we repeat this padded sequence as much as possible within the fixed input length. Finally, we attach zeros at the end to achieve a uniform length across all inputs. The resulting augmented sequence is represented as:

(1) $$S_{\mathrm{repeat}}^{P}=[0,S^{P},0,0,S^{P},0,\ldots ,0]\in R^{2304}$$

The length of $S_{\mathrm{repeat}}^{P}$ is 2304=9×256, where 9 is the empirically chosen number of amino acids in each pocket discussed below, and 256 is the maximum allowed number of pockets considering memory. This approach increases the likelihood of capturing the actual binding site and providing a more comprehensive view of the protein’s structure.

As aforementioned, drugs often bind to a specific segment of a protein sequence, known as a “protein pocket” (Zürcher and Diederich 2008). To fully utilize this prior knowledge, we perform pocket operations after sending $S_{\mathrm{repeat}}^{P}$ into ESM-2, denoted as Pock on consecutive segments of the sequence in $M^{P}$. This operation is formulated as follows:

(2) $$M_{\mathrm{pocket}}^{P}=\left[\frac{1}{9}\sum_{i=1}^{9}m_{i},\ldots ,\frac{1}{9}\sum_{i=2296}^{2304}m_{i}\right]=\text{Pock}(M^{P})=\text{Pock}(\text{ESM}(S_{\mathrm{repeat}}^{P}))$$

where $M^{P}=\left[m_{1},m_{2},\ldots ,m_{2304}\right]$, $m_{i}$ contains embeddings from ESM-2. This process involves dividing the protein sequence into overlapping segments of 9 amino acids each (based on typical binding pocket sizes), extracting features for each segment using ESM-2, and then applying an average operation over these segment features to obtain a single vector representation for each pocket.

It is worth noting that, in addition to the same dimension transformation network as the pretrained drug model, we attached the Adapter (denoted as Ada) in the last layer of ESM-2, which enables the adaptation of the output embedding to the desired distribution, even when only 0.5–8% of the original model parameters are added (Houlsby *et al.* 2019). This makes the transformation of ESM-2 more parameter efficient. After Ada, we applied a FeedForward (Vaswani *et al.* 2017) architecture, denoted as ff, to enhance the learning capacity of the model. The calculation process of the corresponding features can be formulated as follows:

(3) $$M_{\mathrm{ada}}^{P}={\text{Ada}}_{P}(M_{\mathrm{pocket}}^{P})$$

(4) $$M_{ff}^{P}=ff_{P}(M_{\mathrm{ada}}^{P})$$

The PLM embedding of the protein compounds from the FeedForward layer is denoted as $M_{ff}^{P}$. The similar dimensional transformation network with no shared weights is also applied to the features output by the pretrained drug model to standardize the unified vector dimension.

#### 2.4.2 PLM for drug

ChemBERTa-2 (Ahmad *et al.* 2022) is a RoBERTa-like (Liu *et al.* 2019) PLM based on SMILES. It uses up to 77M drug molecules from PubChem (Bolton *et al.* 2008) and builds a basic model suitable for chemical molecular machine learning through self-supervised learning (SSL) training. To obtain the drug molecule’s embedding, we utilize ChemBERTa-2, which encodes it based on the drug’s SMILES sequence. To ensure alignment among drug lengths, we trim excessively long drugs and pad insufficiently long molecules with zeros. This results in the trimmed input drug SMILES $S_{\mathrm{trim}}^{D}\in {\mathbb{R}}^{\Theta_{d}}$ and suitable embeddings of drug compounds for batch training, with $\Theta_{d}=512$ representing the maximum allowed length for a drug sequence. Given the trimmed input drug SMILES, the feature $M^{D}$ is derived from PLM:

(5) $$M^{D}=\text{ChemBERTa}(S_{\mathrm{trim}}^{D})$$

and the FeedForward architecture is used as a projected non-linearity to transform the general feature space:

(6) $$M_{ff}^{D}=ff_{D}(M^{D})$$

### 2.5 Feature extractors for protein and compound

#### 2.5.1 Sequence feature extractor for protein

As we mentioned in Eq. (1), we also use $S_{\mathrm{repeat}}^{P}$ as input sequence for protein feature extractor. The protein feature extractor comprises three layers of 1D convolutional layers (Kiranyaz *et al.* 2021), which transform the protein sequence into a matrix representation within the latent feature space. Each row of the resulting matrix represents the features of an amino acid. Following the concept of word embedding (Levy and Goldberg 2014), we initially create a lookup table with all types of amino acids as keys. By referencing this lookup table, each repetitive protein sequence can be initialized to a corresponding feature matrix ($X_{p}^{FE}$). Subsequently, $X_{p}^{FE}$ is fed into the 1D protein feature extractor, composed of convolutional layers, to extract the local substructure patterns of the protein. By gradually increasing the convolution kernel, the feature extractor can learn the multi-scale local fragment features of the protein. Each layer of the extractor network can be represented as follows:

(7) $$H_{p}^{\left(l+1\right)}=\text{BN}(\text{ReLU}(\text{CNN}(W_{\mathrm{cnn}}^{\left(l\right)},b_{\mathrm{cnn}}^{\left(l\right)},H_{p}^{\left(l\right)})))$$

In the above formula, $W_{\mathrm{cnn}}^{\left(l\right)}$ and $b_{\mathrm{cnn}}^{\left(l\right)}$ denote specific learnable parameter matrices and bias vectors for each layer, ReLU represents a non-linear activation function (Nair and Hinton 2010), BN signifies a parameter-less one-dimensional BatchNorm (Ioffe and Szegedy 2015) operation, $H_{p}^{\left(l\right)}$ represents the lth hidden protein embeddings and $H_{p}^{\left(0\right)}=X_{p}^{FE}⊕M^{P}$, where ⊕ denotes concatenation. The final embedding of the extractor is denoted as $E^{P}=H_{p}^{\left(3\right)}$. After obtaining $E^{P}$, we do the pocket process like Eq. (2) as follows: $E_{\mathrm{pocket}}^{P}=\text{Pock}\left(E^{P}\right)$. The 1D convolution treats the protein sequence as an overlapping multi-mer amino acid sequence, capturing residue-level features composed of 3-, 6-, and 9-mer fragments in a layered manner.

#### 2.5.2 Graph feature extractor for compound

For the drug feature extractor, in order to effectively utilize the connection properties among drug atoms, we transform the trimmed SMILES $S_{\mathrm{trim}}^{D}$ into the corresponding 2D molecular graph, denoted as G. By utilizing the functions encapsulated in the DGL-LifeSci (Li *et al.* 2021) package, we initialize each atom node in G based on its chemical properties. Each atom is assigned a vector comprising integers, representing distinct information from 8 fields: the atom type, the atom degree, the number of implicit Hs, the number of total Hs, the number of formal electrons, the number of radical electrons, the atom hybridization and aromaticity. To ensure consistency in the size of different molecular graphs, we construct virtual nodes filled using zeros for molecules with fewer than $\Theta_{d}$ atom nodes. Consequently, the node feature matrix $X_{d}^{FE}$ of each graph is applied a linear transformation $W_{t}\in R^{D_{d}^{G}\times D_{d}^{FE}}$, converting the integer features within the node feature matrix into real continuous values, denoted as ${X^{\prime }}_{d}^{FE}$. This transformed matrix serves as the input for the drug feature extractor.

The drug feature extractor comprises three layers of GCN (Kipf and Welling 2016) that effectively adapt the structural information of drugs in graph G. GCN is a neural network that extends the convolution operation to graph data structures. Specifically, GCN first gathers the feature vectors of all atoms in the neighborhood, performs an aggregation operation to obtain the ’message’, and then updates the atom features. The neighborhood is defined by the chemical bonds in which the atom participates. Each layer of the extractor network can be represented as follows:

(8) $$H_{d}^{\left(l+1\right)}=\text{GCN}(A,W_{\mathrm{gcn}}^{\left(l\right)},b_{\mathrm{gcn}}^{\left(l\right)},H_{d}^{\left(l\right)})$$

In the above formula, $W_{\mathrm{gcn}}^{\left(l\right)}$ and $b_{\mathrm{gcn}}^{\left(l\right)}$ are the learnable parameter matrix and bias vector of the lth GCN layer, respectively. A represents the adjacency matrix that includes self-connections in graph G, and $H_{d}^{\left(l\right)}$ denotes the lth hidden drug embeddings, with $H_{d}^{\left(0\right)}$ being equal to ${X^{\prime }}_{d}^{FE}$.

We denote the last embedding of the extractor as ${E^{D}=H}_{d}^{\left(3\right)}$. The three-layer GCN structure of the extractor empowers DrugLAMP with the capability to capture information about molecular substructures at various scales.

### 2.6 Contrastive compound-protein pre-training

The 2C2P module enhances DrugLAMP’s generalization ability through multi-scale contrastive learning. This module leverages self-supervised learning techniques to capture meaningful representations from both protein sequences and drug compounds, while simultaneously aligning these representations across modalities.

The rationale for incorporating contrastive learning is three-fold: (i) it enables learning from unlabeled data, which is particularly valuable in drug discovery where labeled data are scarce; (ii) it helps distinguish between interacting and non-interacting drug-protein pairs; and (iii) it encourages learning representations that are invariant to irrelevant transformations, improving model robustness and generalization.

The module consists of three core components. For these components, we formulate their respective loss functions to guide the learning process (Technical implementation details and considerations are provided in the Supplementary Materials):

MLM (Devlin *et al.* 2018) for proteins: This component randomly masks portions of amino acid sequences and tasks the model with predicting these masked elements based on contextual information. By learning to recover masked amino acids, the model develops a deep understanding of protein sequence patterns and their biological properties. The MLM loss function combines cross-entropy losses from two prediction paths, ensuring robust protein representation learning. Given the ground-truth of masked amino acids labels, the loss is computed as:

(9) $$L_{\mathrm{PSS}}\left(S_{\mathrm{repeat}}^{P},M^{P}\right)=\left[CE\left(\mathrm{logit}s_{S},\mathrm{labels}\right)+CE\left(\mathrm{logit}s_{M},\mathrm{labels}\right)\right]/2$$

In the above formula, ${\mathrm{logits}}_{S}$ and ${\mathrm{logits}}_{M}$ denote the predicted probabilities of labels by $S_{\mathrm{repeat}}^{P}$ and $M^{P}$, respectively. CE refers to the cross-entropy function (refer to pseudo code (Algorithm 1 in the Supplementary Materials) for specific implementation details).

SimSiam (Chen and He 2021) for drug compounds: This self-supervised approach creates two augmented views of each drug molecule and processes them through the encoder network to obtain representations. The SimSiam framework uses a predictor network and a stop-gradient operation to prevent representation collapse while maximizing the similarity between different views of the same molecule. This design helps capture robust and transferable molecular features that are invariant to minor structural variations. The supervision loss function is defined as:

(10) $$L_{\mathrm{DSS}}\left(E^{D},M^{D}\right)=[DS(E_{\mathrm{Pred}}^{D},M_{\mathrm{Proj}}^{D^{*}})+DS(E_{\mathrm{Proj}}^{D^{*}},M_{\mathrm{Pred}}^{D})]/2$$

In the above formula, $E_{\mathrm{Pred}}^{D}$ and $M_{\mathrm{Pred}}^{D}$ are obtained by passing $E^{D}$ and $M^{D}$, respectively, through two given ProjNets and the same Predictor. $E_{\mathrm{Proj}}^{D^{*}}$ and $M_{\mathrm{Proj}}^{D^{*}}$ represent the two input features after passing through their respective ProjNets without back-propagation. DS is the negative cosine function that calculates the similarity between different modalities of drugs.

Cross-modal triplet loss: This component aligns drug and protein representations through carefully constructed triplets (anchor protein, positive drug, negative drug). For each protein (anchor), we identify positive drug compounds (known interactions) and negative ones (non-interactions) based on a ground-truth interaction matrix. The extended triplet loss (Deng *et al.* 2020) minimizes the distance between interacting pairs while maintaining a margin-based separation from non-interacting pairs. The margin is dynamically adjusted during training to balance early-stage alignment and later-stage discrimination. Given constructed comparable positive and negative sample pairs, we can compute the triplet margin-distance loss ($L_{CM}$):

(11) $$L_{CM}(a,p,n)=\frac{1}{N_{\mathrm{tri}}}\sum_{i=1}^{N_{\mathrm{tri}}}\text{max}(\text{dist}(a_{i},p_{i})-\text{dist}(a_{i},n_{i})+m,0)$$

In the above formula, a, p, and n represent the anchor, positive, and negative examples, respectively. The lengths of a, p and n are equal and denoted as $N_{\mathrm{tri}}$. dist represents the function that computes pairwise distances between inputs, using the $l^{2}$-norm. The margin m is a value that changes with each epoch and determines the maximum required delta between distances to make $L_{CM}$ zero. At epoch t, the margin is set as follows:

(12) $$m(t)=m_{\max}\cdot (1-\text{tanh}(2\cdot (1-\frac{t\;\text{mod}\;N_{re}}{N_{re}})))$$

These components are integrated directly into DrugLAMP’s main training process, rather than requiring separate pre-training. Building upon the success of cross-modal learning approaches such as CLIP (Radford *et al.* 2021) and DeCLIP (Li *et al.* 2021), our 2C2P module extends these principles to address the specific challenges of drug–target interaction prediction. While CLIP focuses on image–text alignment, we adapt and enhance its methodology for the drug–protein domain by incorporating additional self-supervised learning objectives.

The integration of these three components creates a comprehensive learning framework. MLM ensures meaningful protein sequence representations by capturing local and global amino acid patterns, while SimSiam develops robust molecular representations that are consistent across different views of the same compound. The cross-modal triplet loss then aligns the learned representations across modalities while maintaining discriminative power. This multi-component design enables DrugLAMP to learn biologically meaningful representations that generalize well to unseen drug–protein pairs.

### 2.7 Pocket-guided co-attention

Existing DTI methods often fail to capture the biological details of drug–protein interactions due to the data heterogeneity between protein sequences and drug SMILES data. These methods typically rely on simple concatenation or post-fusion techniques. To address this limitation, we propose a more sophisticated feature aggregation strategy that directly models the interaction between pairwise drug-protein token-level features, regardless of whether these features originate from a pretrained model or a feature extractor. Simulation as the standard Attention mechanism for associating image and text embeddings in the visual question answering problem (Chen *et al.* 2021), we designed PGCA. Since the protein’s binding pocket is far larger than the drug molecule on the volume scale, PGCA uses protein’s pocket embedding to guide the aggregation of drug molecular features, making it a rough clustered pocket-guided drug feature embedding. We assume that features obtained using the same method may have similar characteristics. Therefore, fusing the features of drug–protein pairs obtained using the same method can better capture the matching information between drugs and proteins.

The PGCA module uses protein pocket information to guide the attention mechanism on drug features. It first computes attention weights based on the similarity between protein pocket embeddings and drug atom embeddings. These weights are then used to create a weighted sum of drug atom features, effectively highlighting the most relevant parts of the drug molecule for interaction with the specific protein pocket.

During the PGCA process, we perform feature fusion based on the feature extraction method, dividing them into two groups: $M_{ff}^{P}$ with $M_{ff}^{D}$, and $E_{\mathrm{pocket}}^{P}$ with $E^{D}$. To make the model focus on more valuable information, we apply multi-head attention (Vaswani *et al.* 2017), denoted as MHA, which allows us to project $F_{\mathrm{coattn}}^{M}$ ($F_{\mathrm{coattn}}^{E}$) to different spaces to highlight more critical information.

The following formula is followed:

(13) $$\begin{array}{c} F_{\mathrm{coattn}}^{M}=\text{CoAtten}(M_{ff}^{P},M_{ff}^{D})\\ =\text{softmax}(\frac{W_{q}M_{ff}^{P}M_{ff}^{D}{}^{⊺}W_{q}{}^{⊺}}{\sqrt{d_{k}}})W_{v}M_{ff}^{D}\\ F_{\mathrm{mixed}}^{M}=\text{Norm}(\text{MHA}(F_{\mathrm{coattn}}^{M})⊕F_{\mathrm{coattn}}^{M})\\ F_{\mathrm{coattn}}^{E}=\text{CoAtten}(E_{\mathrm{pocket}}^{P},E^{D})\\ =\text{softmax}(\frac{W_{q}E_{\mathrm{pocket}}^{P}E^{D}{}^{⊺}W_{k}{}^{⊺}}{\sqrt{d_{k}}})W_{v}E^{D}\\ F_{\mathrm{mixed}}^{E}=\text{Norm}(\text{MHA}(F_{\mathrm{coattn}}^{E})⊕F_{\mathrm{coattn}}^{E}) \end{array}$$

During the MHA process, we use $F_{\mathrm{coattn}}^{M}$ ($F_{\mathrm{coattn}}^{E}$) for both key, value and query.

On the one hand, PGCA can effectively use pocket-guided drugs to achieve the purpose of multi-modal feature alignment; on the other hand, with the help of the Attention mechanism, we can visualize important hidden pockets of drug-protein interactions learned by the model.

### 2.8 Paired multi-modal attention

This module method is mainly derived from the multi-modal fusion problem developed in the field of computer science. The early multi-modal fusion module is mainly used for the alignment of images and other modalities. In the medical multi-modal fusion problem, there are also many works dedicated to multi-modal fusion problem with different methods, such as applying attention-based layers.

We designed and developed a multi-modal attention fusion module to fuse the mixed features of the drug–protein pairs after alignment. To consider both the general nature of the pretrained model’s representation and the specific characteristics of the feature extractor, we use an attention mechanism to weight the feature representations of the two, especially to emphasize the differences between the shallow representations of drugs or proteins in the training data. Since we assume that features obtained using different methods may have their own unique advantages, we adopt a more restrained fusion approach to preserve the uniqueness of features obtained from different methods. After obtaining $F_{\mathrm{mixed}}^{E}$ and $F_{\mathrm{mixed}}^{M}$ by PGCA, we apply the paired multi-modal attention layer (Fig. 1c), denoted as PML, to fuse features restrainedly with retaining unique characteristics from different methods:

(14) $$\begin{array}{c} F^{E}=\text{PML}{\left(F_{\mathrm{mixed}}^{E},F_{\mathrm{mixed}}^{M}\right)}_{E}+F_{\mathrm{mixed}}^{E}\\ F^{M}=\text{PML}{\left(F_{\mathrm{mixed}}^{E},F_{\mathrm{mixed}}^{M}\right)}_{M}+F_{\mathrm{mixed}}^{M} \end{array}$$

Then we send $F^{E}$ and $F^{M}$ into multi-layer perceptron, denoted as MLP, to expand the learning space, and obtain the final feature by the attention mechanism. The calculation process is followed:

(15) $$\begin{array}{c} F_{\mathrm{final}}^{E}=\text{MLP}(\text{Norm}(F^{E}))+F^{E}\\ F_{\mathrm{final}}^{M}=\text{MLP}(\text{Norm}(F^{M}))+F^{M}\\ F_{\mathrm{final}}=\text{Attention}(F_{\mathrm{final}}^{E},F_{\mathrm{final}}^{M}) \end{array}$$

During the calculation of PMMA process, we symmetrically compute the features obtained from two different methods. This allows us to combine the generalization capability of the PLM with the specificity of the extractor, resulting in a final set of features.

### 2.9 Model training and evaluation

For our primary evaluation, we focus on two key metrics: AUROC and AUPRC. These metrics are particularly effective for evaluating binary classification problems, which is common in drug-target interaction prediction tasks. AUROC provides a measure of the model’s ability to distinguish between classes, while AUPRC is particularly informative when dealing with imbalanced datasets. Five experiments were conducted for each distinct dataset split using different random seeds. The interpretive and visualization part utilized the best-performing model trained on the BioSNAP random split.

## 3 Results

The DrugLAMP framework, as illustrated in Fig. 1, predicts drug–target interactions through a multi-step pipeline that encodes drug–target data to multiple modalities, applies attention-based feature guidance, and fuses multi-modal information for comprehensive interaction prediction. To enhance performance and generalizability, the framework incorporates self-supervised and contrastive learning module. The subsequent section provides a detailed exposition of each module.

### 3.1 Performance comparison in standard scenario

**Figure 2.**
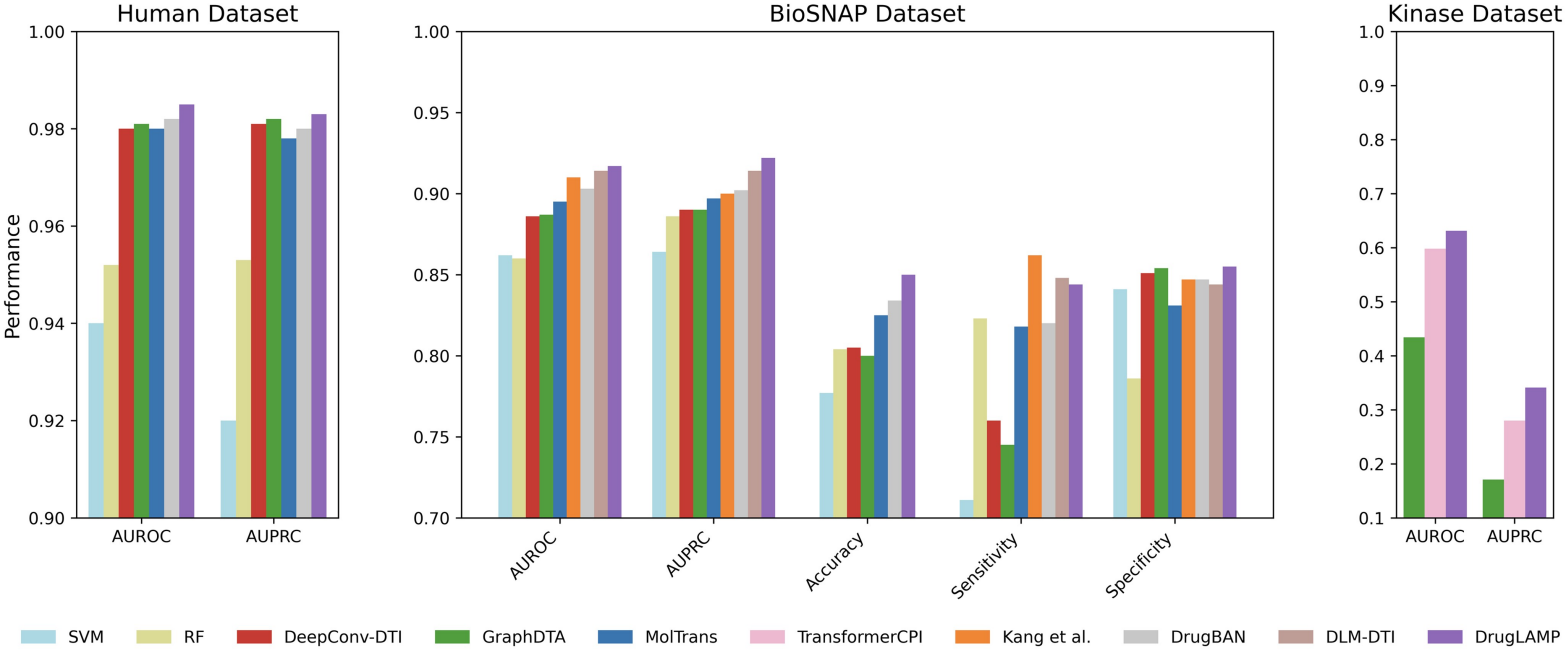
Common performance comparison on the three datasets in standard scenario. The 'Performance' depicted in the figure represents the average value of the respective metric derived from five experiments conducted with different random seeds.

We compared DrugLAMP with nine other baseline methods in a random split setting: support vector machine (SVM) (Cortes and Vapnik 1995), random forest (RF) (Ho 1995), DeepConv-DTI (Lee *et al.* 2019), GraphDTA (Nguyen *et al.* 2021), MolTrans (Huang *et al.* 2021), TransformerCPI (Chen *et al.* 2020), Kang *et al.* (2022), DrugBAN (Bai *et al.* 2023), and DLM-DTI (Lee *et al.* 2024). Given that this setting is a typical conventional setting, we opted not to include the cross-model contrastive learning in 2C2P module in our model for this scenario. Figure 2 displays the results on the Kinase (Chen *et al.* 2020), BioSNAP (Zitnik *et al.* 2018), and Human (Liu *et al.* 2015) datasets. DrugLAMP surpasses other existing methods, including the newly published benchmark DLM-DTI based on PLMs, in terms of area under the receiver operating characteristic curve (AUROC) and area under the precision–recall curve (AUPRC). Based on the data presented in the table, DrugLAMP outperforms other methods significantly in the human dataset, showing improvements of 0.305% in AUROC and 0.102% in AUPRC. On the BioSNAP dataset, DrugLAMP achieves state-of-the-art performance across four metrics, including AUROC and AUPRC. It is worth mentioning that Kang *et al.* another method based on PLMs, excels in the sensitivity metric. Regarding the kinase dataset, we observed enhancements of 5.518% and 21.786% in AUROC and AUPRC metrics, respectively.

In Fig. 2, we present AUROC and AUPRC metrics for all three datasets (BioSNAP, Human, and Kinase) as these are our primary evaluation metrics. For the BioSNAP dataset, we provide additional metrics (Accuracy, Sensitivity, and Specificity) to offer a more comprehensive view of the model’s performance. We chose to present these additional metrics for BioSNAP because it is the largest and most diverse of the three datasets, making it particularly suitable for in-depth analysis. Furthermore, the model used for interpretive and visualization studies (as described in Section 2.9) was trained on the BioSNAP random split, which motivated us to provide a more detailed performance breakdown for this dataset. We have included a supplementary table (Table S5) in the Supplementary Materials that presents raw data sources for each of the three datasets.

The results indicate that under an ordinary random data split, the PLMs-based methods can glean more feature information than methods relying solely on small data-driven and manually defined features. The compound features produced by the feature extractor and PLM are aligned with the pocket feature space of proteins using the PGCA module. Thanks to the PMMA module employed by DrugLAMP, the performance of the test can be further improved because it can integrate the complementary information between different modalities.

### 3.2 Performance comparison in two real-world scenarios

Performing DTI prediction under a conventional random dataset split setting is fairly straightforward. However, this split setting diverges from actual pharmaceutical scenarios. Consequently, we are committed to tackling more challenging scenarios. These scenarios require the model’s capacity for simulating reality, necessitating its adaptation to test datasets that differ in data type and distribution from the training set in order to achieve favorable performance. We firmly believe that these tasks represent a broad sample of real-world drug discovery scenarios, setting them apart from conventional random split tasks.

Two different splits are used. In the ’cold-start’ splitting, the validation set and the test set are initially selected, followed by the removal of the remaining data. The training set is then constructed by pairing in the remained drugs and targets. In the ’cluster-start’ splitting, the dataset is split into two non-overlapping domains based on clustering results. These two split settings are formulated to enable exploration of real-world scenarios. To facilitate this, we incorporate the cross-model contrastive learning in 2C2P module into our model.

Table 1 illustrates the performance evaluation conducted on the ’cold-start’ split of the Human dataset and the ’cluster-start’ split of the BindingDB and BioSNAP datasets. Compared to the conventional random split, the ’cold-start’ split removes identical drugs/targets from the test set, resulting in varying degrees of performance decline across all DTI models. Furthermore, the ’cluster-start’ split imposes additional specifications, necessitating distinct distribution of test and training data, resulting in a more substantial performance decline. Despite these constraints, it is worth noting that DrugLAMP consistently outperforms other state-of-the-art models. For the ’cold-start’ split of the Human dataset, DrugLAMP achieves superior performance with an AUROC of 1.18% and an AUPRC of 2.52%. Similarly, for ’cluster-start’ splits of the BindingDB and BioSNAP datasets, DrugLAMP exhibits 7.62%/8.04% higher AUROC values, and 5.76%/4.35% better AUPRC values, respectively. These results demonstrate DrugLAMP’s remarkable robustness, excelling in both ideal and real-world scenarios. This implies that DrugLAMP’s success does not solely rest on the training data biases in achieving state-of-the-art performance in ideal scenarios. Furthermore, by incorporating the 2C2P module into the model, it proactively tackles challenges encountered in numerous real-world drug discovery scenarios. This module guides the PLM to adapt and excel in zero-shot prediction tasks within real-world settings. As a comparison, experiments were also conducted using a model without the 2C2P module. The results in Table 1 demonstrate the indispensable role of the 2C2P module in enhancing DrugLAMP’s generalization capabilities in real-world scenarios.

**Table 1.** Performance comparison on three datasets in real-world scenarios (statistics over five random runs).

Models	Human_Cold_	BindingDB_Cluster_	BioSNAP_Cluster_
SVM	(0.691, 0.629)	(0.535, 0.491)	(0.624, 0.626)
RF	(0.732, 0.670)	(0.564, 0.503)	(0.614, 0.604)
DeepConv-DTI	(0.820, 0.780)	(0.539, 0.474)	(0.627, 0.632)
GraphDTA	(0.816, 0.770)	(0.530, 0.467)	(0.637, 0.644)
MolTrans	(0.804, 0.767)	(0.536, 0.477)	(0.635, 0.629)
DrugBAN	(0.850, 0.794)	(0.604, 0.556)	(0.684, 0.736)
DrugLAMP_CM_ w/o 2C2P	(0.857, 0.795)	(0.643, 0.587)	(0.739, 0.760)
DrugLAMP_CM_	**(0.860, 0.814)**	**(0.650, 0.588)**	**(0.739, 0.768)**

Values are presented in the format of (AUROC, AUPRC). Bold numbers indicate the best performance achieved on the corresponding dataset. w/o: without.

### 3.3 Ablation study to support the proposed modules

Ablation studies were also conducted on DrugLAMP’s various modules and multi-modal paradigm on the Human dataset in a cold-start split setting, confirming their contributions to our model’s outstanding performance, as detailed in Table 2. We compared DrugLAMP with six ablation models. Initially, we examined an ablation model that excludes the contrastive loss (Ablation I (w/o 2C2P)). Subsequently, Ablation II (w/o PGCA) was developed to illustrate that the enhancement of DrugLAMP results from the attention structure understanding the DTI mechanism. Furthermore, we adjusted the multi-modal input to remove PLMs and introduced Ablation III (w/o PLM) while utilizing the same feature extractors. We also attempted to eliminate the feature extractors as part of Ablation IV (w/o FE). Additionally, we conducted Ablation V (w/o repeat) to illustrate the importance of repeating the protein sequence and Ablation VI (w/o PMMA) to reflect the contribution of fusing features from different extraction methods to model performance.

**Table 2.** Module ablation studies on human dataset in cold-start split (statistics over five random runs).

Variants	AUROC	AUPRC
DrugLAMP_CM_ w/o PLM	0.829	0.761
DrugLAMP_CM_ w/o FE	0.835	0.765
DrugLAMP_CM_ w/o repeat	0.841	0.749
DrugLAMP_CM_ w/o PGCA	0.837	0.767
DrugLAMP_CM_ w/o PMMA	0.846	0.781
DrugLAMP_CM_ w/o 2C2P	0.857	0.795
DrugLAMP_CM_	**0.860**	**0.814**

Bold numbers indicate the best performance. w/o: without.

The ablation models were assessed on the Human dataset using a cold-start split, and the performance is detailed in Table 2. Based on the findings from the table, four key observations were made: (i) The absence of contrastive loss diminishes performance, highlighting the significance of 2C2P in improving learned representations of multi-modal features; (ii) The superiority of DrugLAMP over ablation II and VI indicates that the enhancement is attributed to attention accurately mimicking the DTI structure and correctly preserving and integrating complementary multi-modal information; (iii) Compared to not using the repeat operation, ablation V shows that allowing the model to consider multiple potential binding sites simultaneously can enhance the model’s comprehensive understanding of DTI; (iv) the decrease in performance upon removing PLMs compared to a standard DTI prediction network underscores the superiority of DrugLAMP. However, it is noteworthy that, as demonstrated by ablation IV, traditional feature extractors also form the foundation for accurately predicting DTI.

These findings provide strong empirical support for the design choices in DrugLAMP and demonstrate how each module contributes to the model’s overall performance.

While the improvement introduced by some module may seem modest in terms of percentage, it translates to a significant enhancement in predictive accuracy, especially considering the already high performance of our base model. This improvement is particularly valuable in the context of drug discovery, where even small increases in accuracy can lead to substantial time and cost savings in the drug development process.

### 3.4 DrugLAMP revealing the adverse event mechanisms of known drugs

A single drug can interact with hundreds of protein targets associated with adverse events (ADEs). However, identifying the unintended ’off-targets’ that can predict adverse events is challenging because some may not correlate with conventional molecular metrics (Cheng *et al.* 2018, 2019). Therefore, we evaluated DrugLAMP’s performance by predicting a drug–target–ADEs network for irritable bowel syndrome and decompensated heart failure, curated from a recent study (Zeng *et al.* 2020).

**Figure 3.**
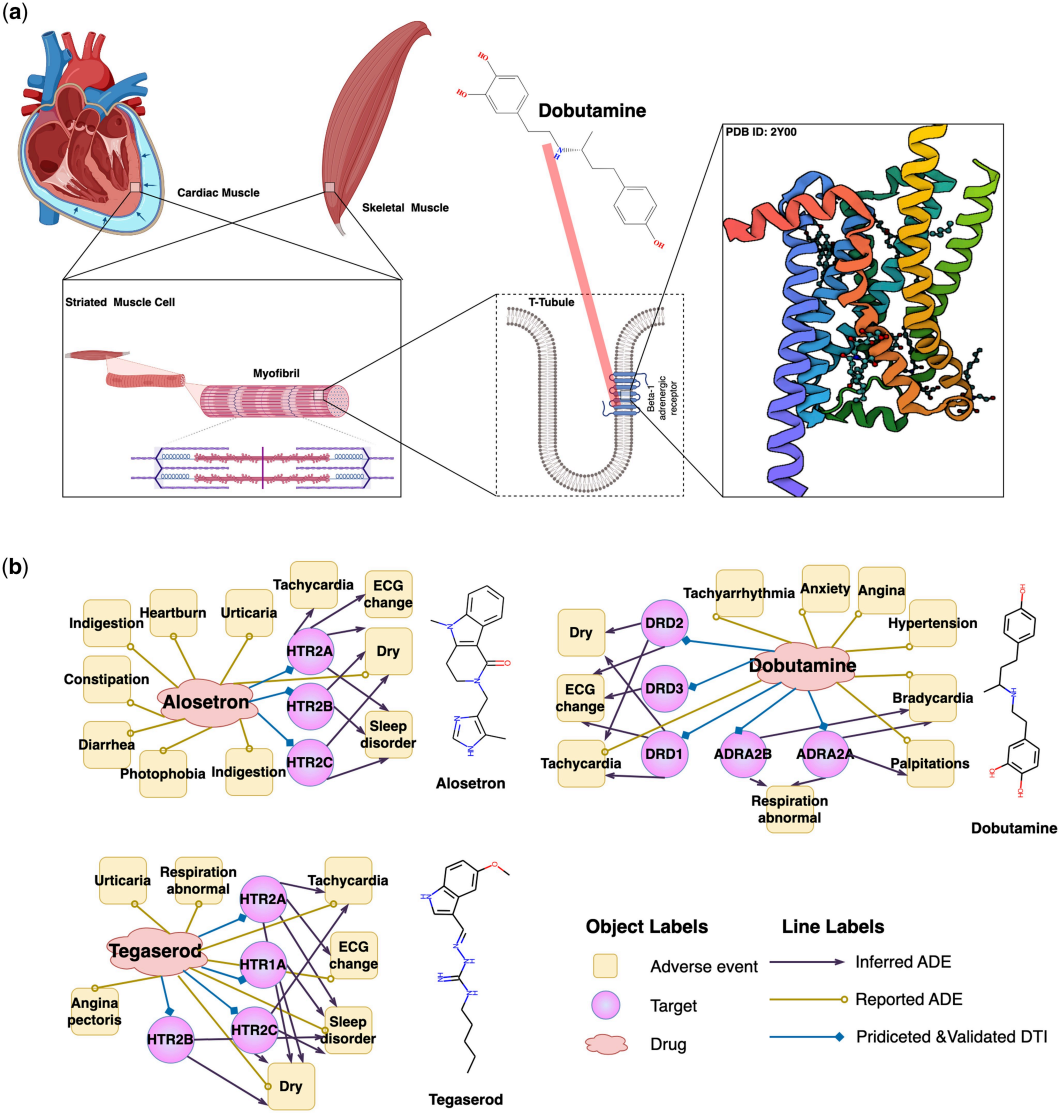
Schematic diagram of the mechanism of Dobutamine's primary activity and three drug-target-ADEs networks consisting of drugs and their off-targets inferred by DrugLAMP. (a) Schematic diagram of the on-target mechanism of dobutamine. Created with Biorender.com. (b) Drug–target-ADE networks about three approved drugs. Drug–target interactions were predicted by DrugLAMP. Drug–ADEs were collected from metaADEDB. Target–ADEs were inferred with data from DrugBank, DrugCentral, and a recent study.

Dobutamine is a direct-acting inotropic agent, and its primary activity results from stimulating the beta1-adrenoceptors of the heart (Fig. 3a). Nevertheless, several ADEs are associated with dobutamine treatment (Cheng *et al.* 2013). Bradycardia is one such event. We predict through DrugLAMP that ADRA2A and ADRA2B are off-targets and posit that they are closely related to bradycardia (Fig. 3b). This inference can be confirmed through genetic and computational analyses (Kurnik *et al.* 2006, Staessen *et al.* 2008, Tikhonoff *et al.* 2008, Zeng *et al.* 2020). Tegaserod and alosetron were initially used to treat irritable bowel syndrome, but they have been withdrawn due to cardiovascular complications associated with off-targets HTR1A and HTR2B, as inferred by our model. Studying ADR mechanisms can provide insights for the development of new drugs, even for other diseases. For example, the recent discovery of 2-AR agonists, some of which can be used clinically, may significantly improve the clinical efficacy of cancer immunotherapy (Zhu *et al.* 2023).

The above analysis suggests that our model can accurately infer the drug–target–ADEs network. This could help prevent catastrophic drug toxicities frequently identified only after fatal incidents in clinical settings and facilitate the prioritization of safer molecules for pre-clinical development.

### 3.5 Attention heatmap uncovers the underlying patterns of drug–target binding

**Figure 4.**
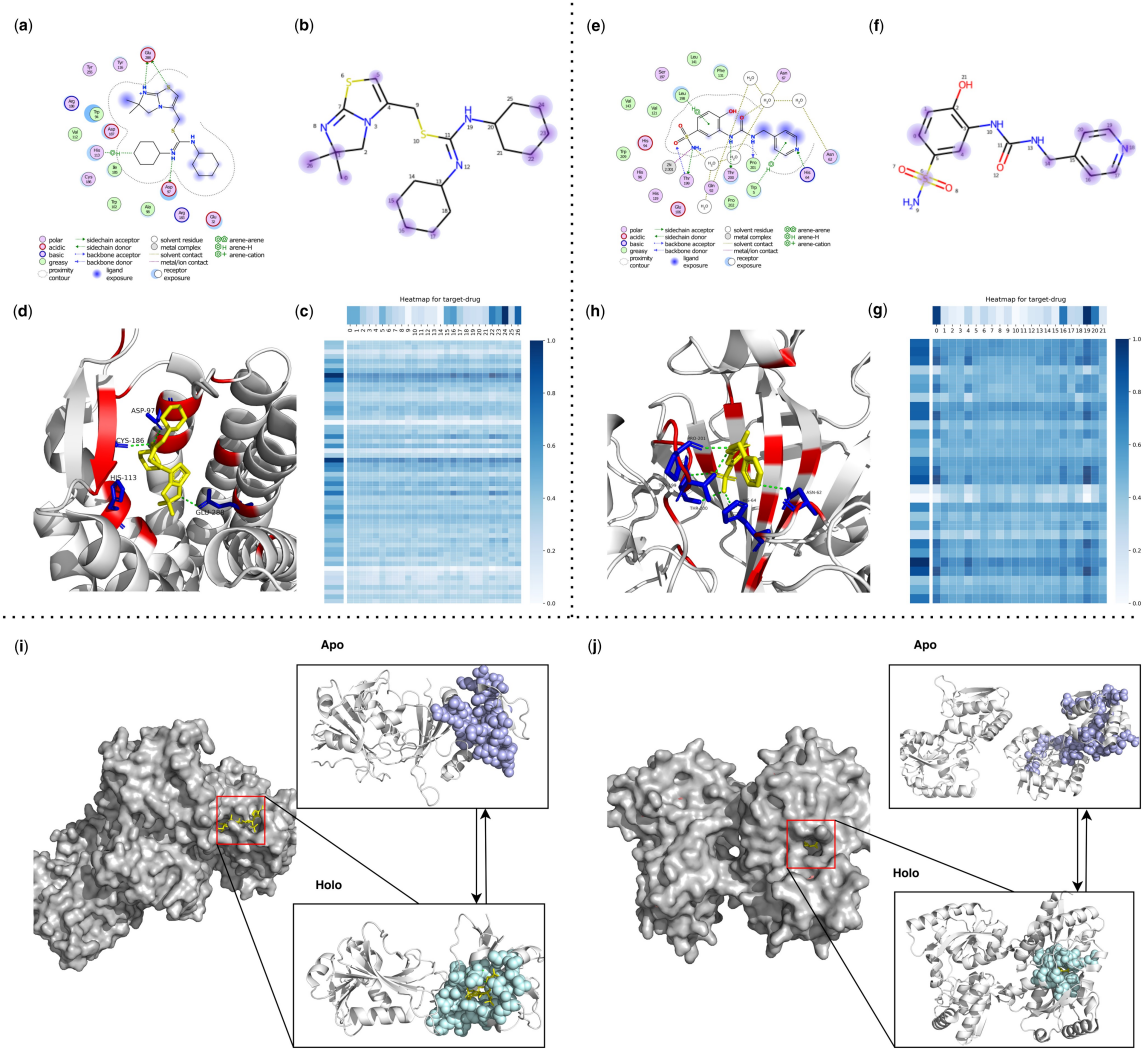
Visualization of ligands, binding pockets, DrugLAMP attention heatmaps and cryptic pockets for interpretability and generalization study. (a, e) Ligand interactions for IT1t in 3ODU and 3 g in 8FQX created by software molecular operating environment (Vilar *et al.* 2008) as a comparative reference for the prediction results of DrugLAMP. (b, f) We highlight top-8 atom as blue dots according to attention bar of drug in c, g, and the diameter of these dots is positively correlated with the degree of attention they receive. (c, g) Attention map for residues of target and atoms of ligand. For target, every nine adjacent residues are merged into a cluster to participate in prediction, ensuring that the predicted key residues are concentrated near one pocket rather than scattered. (d, h) 3D interactions diagram created by PyMol. The residues involved in forming pockets are set to color red. The key residues involved in the interaction are set to color blue and form sticks. (i) Loop motion. (j) Interdomain motion.

To demonstrate the strong interpretability of our model, we selected one representative drug from each of the two most common types of receptors (membrane proteins and enzymes) for analysis. DrugLAMP generates multi-head attentions for both the target and the ligand, enabling the calculation of weights assigned to target residues and ligand atoms (Fig. 4). These weights facilitate the identification of the relative importance of these components in the prediction process.

3ODU, a structure depicting the CXCR4 chemokine receptor (G protein coupled) in complex with the small molecule antagonist IT1t, plays a specific role in cancer metastasis and HIV-1 infection. For IT1t, both cyclohexane rings snugly occupy small sub-pockets (Fig. 4d), establishing contacts with CXCR4 (Fig. 4a). Atoms 22–24 and 15–17 of the IT1t bear high weights in the drug attention analysis, indicating their exposure to the solvent (Fig. 4b). Atom 26 and Atom 5 also received strong attention, which is consistent with the real interaction (Fig. 4a).

8FQX is a structure consisting of CA II (the most prevalent carbonic anhydrase isoform) and the ligand 3 g. Dysregulation of CAs is associated with numerous diseases, including cancers, spurring the development of CA inhibitors (e.g. 3 g) a widely studied topic. For 3 g, atoms 14, 16, 19, and 20 are exposed to the ligand (Fig. 4e) and have been predicted by DrugLAMP (Fig. 4f). 3 g also shares a hydrogen bond with H64 through atom 18, a key interaction point captured by our model (Fig. 4f).

The aforementioned investigation showcases that our proposed model can infer the contributions of individual residues in the target and atoms in the drug to the formation of DTI through the attention weights it learns. This feature demonstrates the interpretability of our model, allowing us to elucidate the mechanism of drug action and gain valuable insights for future drug optimization endeavors.

### 3.6 DrugLAMP accurately predicts cryptic pockets with multiple types of conformational change

Identifying cryptic pockets represents a promising opportunity to expand the scope of drug discovery. Some target proteins are currently undruggable due to the lack of pockets in their ligand-free structures. Existing models that consider only the degree of structural coincidence between ligand-free protein and molecules are ineffective in predicting cryptic pockets. To assess the capacity of DrugLAMP in detecting sites of cryptic pocket formation with the emergent abilities of PLM, we chose two apo-holo protein structure pairs for analysis. These represent two common and distinctive types of conformational changes that lead to cryptic pocket formation from the PocketMiner dataset (Meller *et al.* 2023).

In the left holo form (PDB ID: 2W9S), representing loop motion, loops in the left apo form (PDB ID: 2W9T) separate and converge to cover the incoming ligand (Fig. 4i). The right holo form (PDB ID: 5OTA) depicts inter-domain motion, where a larger inter-domain distance can accompany a more substantial pocket opening (Kumar *et al.* 2020). The shift from the right apo form (PDB ID: 4P0I) to the right holo form (PDB ID: 5OTA) leads to larger pocket openings, which facilitate the formation of ligand-binding sites (Fig. 4j).

By taking the sequence of the apo form protein and the Simplified Molecular Input Line Entry System (SMILES) of the ligand as input, even if the protein structure in apo state and the ligand do not match, DrugLAMP still predicts a pocket. This unexpected performance is attributed to using PLM to characterize various properties of proteins and drugs, rather than relying solely on structural adaptation as the only feature.

## 4 Discussions

Prior to this work, it was unclear how to utilize multi-modal PLMs for DTI prediction. In this study, we introduce DrugLAMP, a framework that incorporates multi-modal information fusion for DTI prediction.

Our novel model, DrugLAMP, uses the advanced molecular PLM ChemBERTa-2 and protein PLM ESM-2 to enhance the molecular graph network and protein feature extractor trained on DTI paired data. We use attention mechanism-based modal fusion modules PMMA and PGCA to effectively manage the multi-modal properties of the DTI problem by sequentially fusing the four input modalities. Additionally, to adapt to real scenarios of computer-simulated drug discovery, we designed the 2C2P module. Through effective contrasts at multiple scale levels, comprehensive experiments have proven that DrugLAMP can accurately predict DTI binding in real scenarios. The potential of DrugLAMP was further demonstrated by constructing drug-side effect maps and predicting cryptic pockets.

The DTI problem is a critical issue in computer-aided drug discovery. This study focuses on leveraging data-based DTI using protein sequences, drug graphs, and PLM embeddings as input. Considering the vast amounts of data utilized during PLM training, we posit that multi-modal inputs can provide complementary 1D, 2D, and 3D perspectives. Rapid DTI prediction based on comprehensive multi-scale information could help reduce the cost of drug research and development, and contribute to the advancement of the human health industry.

While we use ESM-2 and ChemBERTa-2 as PLMs to extract embeddings, the DrugLAMP framework is suitable for any advanced PLM capable of extracting rich information, which enables the incorporation of the latest advancements in PLM. We anticipate that further extension of our ideas to more advanced PLMs could yield improved performance in the future. Lastly, this study investigates performance in different real-world scenarios. Given the continuous emergence of annotated data and new drugs, exploring incremental optimization of DrugLAMP is our next objective.

In summary, the integration of multi-modal contrastive learning modules into PLM transfer is viable and holds significant potential in drug discovery problems. The pretraining process of PLMs based on massive data effectively resolves the dilemma of limited DTI data. The multi-modal contrastive learning method provides comprehensive insights into drug-target combinations. We have open-sourced our code, which can be tailored to individual research needs.

## Supplementary Material

btae693_Supplementary_Data

## Data Availability

The experimental data utilized in this study can be accessed at https://github.com/Lzcstan/DrugLAMP/tree/main/datasets. All data employed in this study originate from publicly available sources. The BindingDB (Gilson *et al.* 2016) source is accessible at https://www.bindingdb.org/bind/index.jsp. The BioSNAP (Zitnik *et al.* 2018, Huang *et al.* 2021) source is accessible at https://github.com/kexinhuang12345/MolTrans/tree/master/dataset/BIOSNAP/full_data. The Human (Liu *et al.* 2015) source can be downloaded at https://github.com/lifanchen-simm/transformerCPI/blob/master/Human%2CC.elegans/dataset/human_data.txt. Additionally, the Kinase (Chen *et al.* 2020) source can be found at https://github.com/lifanchen-simm/transformerCPI/tree/master/data.
