# Supplementary material for: Accurate and transferable drug–target interaction prediction with DrugLAMP
Source: Bioinformatics. 2024 Nov 21;40(12):btae693. doi: 10.1093/bioinformatics/btae693 (PMC11629708; doi:10.1093/bioinformatics/btae693)
Supplement: btae693_Supplementary_Data [file btae693_supplementary_data.pdf]

Accurate and Transferable Drug-Target Interaction Prediction with DrugLAMP
Supplementary Materials

Zhengchao Luo[1], Wei Wu[1], Qichen Sun[2], Jinzhuo Wang[1#]

1 Department of Big Data and Biomedical Artificial Intelligence, College of Future Technology, Peking University, Beijing, 100871, China;

2 School of Mathematical Sciences, Peking University, Beijing, 100871, China.

# Correspondence should be addressed to: wangjinzhuo@pku.com

Implementations Details

Common notations and abbreviations

Tab. S1 contains the important notations and abbreviations used in our study. Other symbols that are trivial or do not play a significant role in the calculation and framework have been omitted.

Dataset settings

In order to effectively evaluate the method of computer-aided DTI prediction task, we split the datasets, and keep a part of the unknown data during training and verification as the test set. The quantities of proteins, drugs, and interactions for each dataset are detailed in Tab. S2. We have conducted thorough experiments on the existing methods, identifying the optimal model through a comparison of classification prediction metrics on the validation set. Subsequently, we documented the performance metrics of the superior model on the test set to ascertain the precise evaluation of the method within the designated dataset under specific split strategy.

There are two types of evaluation experiments, one is the random split evaluation that is usually used for algorithm comparison, and the other is the evaluation of splits that simulate real drug discovery scenarios. Among them, the random split evaluation divides the data set into training, verification and test sets according to the ratio of 70%, 10%, and 20%. In order to simulate real drug discovery scenarios, we propose two split schemes with different emphases: cold-start and cluster-start splits.

In real drug development scenarios, it is often necessary to predict the interaction of new protein targets or newly developed drugs. We evaluate the generality of the method in this case through cold-start splits, where any drug or target in the test set does not appear in the training set. Since there are no molecules identical to the drugs or targets in the test set during the training, better performance indicates the model's ability to learn the role of functional groups of molecules or important residues of targets in binding. Cold-start can also effectively alleviate the overoptimism caused by the molecular bias in the dataset. We performed cold-start experiments in the Human dataset, as previous research (Chen *et al.*, 2020) identified drug bias in the data. We randomly selected 5% and 10% DTI pairs as the validation and test sets, then filtered the remaining DTI pairs containing any of the aforementioned drugs or targets in the training set.

For cluster-start splits, which ensure test set molecules belong to different statistical distributions than the training set, we employed single-linkage clustering following the strategy in (Bai *et al.*, 2023). We specifically chose single linkage clustering for several key reasons: (1) it guarantees that the distance between any two samples from different

clusters exceeds a predefined threshold, which is essential for creating distinct chemical/protein spaces; (2) its tendency to form well-separated clusters aligns with our goal of simulating real-world drug discovery scenarios where test compounds/proteins come from different chemical/structural spaces than training data; and (3) its demonstrated effectiveness in identifying distinct structural families in drug-target interaction data (Bai *et al.*, 2021).

The clustering process utilized ECFP4 (extended connectivity fingerprint, up to four bonds) fingerprint (Rogers and Hahn, 2010) for drugs and pseudo-amino acid composition (PSC) (Cao *et al.*, 2013) for targets. After clustering, 60% of the drug and target clusters were randomly selected as source domain data, with the remaining pairs designated as target domain data, ensuring no distribution overlap between domains. The training set comprised all labeled source domain data and 80% unlabeled target domain data, while 20% labeled target domain data served as the test set. Detailed data split information is provided in Tab. S3. While cold-start split simulates the situation where the drug or target interaction relationship has not been experimentally determined during drug development and evaluates the model's generalization to novel molecules, cluster-start assesses the model's ability to align data with different distributions on more rigorous unseen data, presenting a greater challenge to model generalization.

For the BindingDB and BioSNAP datasets, we employed single-linkage clustering with binary ECFP4 features for drugs and integral PSC features for proteins to simulate real-world scenarios, using Jaccard and cosine distances for pairwise measurements. The clustering ensured sufficient separation between clusters. From the clustering results, we randomly selected 60% of drug clusters and 60% of protein clusters as source domain data, with pairs from remaining clusters forming the target domain data. Tab. S4 presents the sample counts in the ten largest clusters from the clustering results.

Frequencies of drug-target for different scenarios

We demonstrated the frequency of different drugs and targets in positive samples in the dataset under different scenarios. Subfigure a in Fig. S1 represents the situations in three real-world scenarios, while subfigure b illustrates the statistical data under standard scenarios. The results in Fig. S1 indicate that there are instances of a small number of drugs/targets appearing multiple times across different data splits, with Binding DB showing this trend prominently, supporting our use of PLM to introduce extended unlabeled data. Kinase exhibits the least bias, making it one of our choices for the standard scenarios.

Method selection rationale and ethical considerations

Following the frequency analysis shown in Fig. S1, we implemented a principled

approach for method selection in the Kinase dataset evaluation. Our selection strategy was guided by both scientific rigor and ethical considerations. Scientifically, we prioritized methods developed after the dataset's introduction in 2020 (Chen *et al.*, 2020), as the Kinase dataset specifically addresses the challenges of integrating various biological activity scores (IC_{50}, K_i, and K_d) and screening for compounds and proteins with sufficient interaction data. This temporal consideration ensures fair comparison with methods designed with awareness of these specific challenges.

From an ethical perspective, we believe it is crucial to respect peer researchers' contributions by evaluating their methods within their intended experimental contexts. Applying methods to significantly different settings without proper adaptation could potentially misrepresent their original contributions and capabilities. Therefore, our selection criteria encompassed: (1) temporal relevance - methods published after 2020, (2) architectural diversity - including both graph-based and attention-based approaches for comprehensive comparison with PLM-based DrugLAMP, and (3) experimental context alignment - ensuring methods were evaluated on the Kinase dataset in accordance with their original intended settings.

Technical Details of 2C2P Module

Building on the idea of extending the CLIP method, which was originally proposed to address the association mining problem between images and text, we expand the classic Triplet-Loss by incorporating a margin-scheduled distance function to facilitate association learning across intra-, inter-, and cross-modalities in DTI. Compared to a single CLIP supervision method, 2C2P introduces two additional SSL methods: SimSiam for drug compounds and MLM for proteins, providing more comprehensive information.

For the protein sequence processing, alignment between features from the protein extractor and embeddings from the pretrained model has already been achieved. Therefore, instead of concerning ourselves with aligning dimensions from two different encoding methods, we aim to enrich their patterns and improve encoding efficiency through the MLM approach. The variables entering the protein self-supervised learning module are processed according to Eq. (9) in the main text. During implementation, we randomly mask portions of amino acids in the protein sequence based on given probabilities, and the model predicts these masked positions using surrounding context.

Regarding the features from the drug molecule extractor and the embeddings from the pretrained model, due to the specific nature of the graph structure with virtual nodes, the alignment between features after multiple message-passing steps and embeddings from PLM is not strictly preserved. When comparing the two, it is necessary to compare patterns at the molecular level. Therefore, we employ the SimSiam method as described

by Eq. (10) in the main text.

For cross-modality contrastive learning, triplets of training batches are used instead of pairs. These triplets consist of an anchor, a positive example, and a negative example. The objective is to minimize the distance between the anchor and positive examples while maximizing the distance between the anchor and negative examples. In the DTI setting, triplets are derived from a batch as follows: known interacting drug-target pairs are marked as positive (T, D^{+}), other unknown or known non-interacting pairs are marked as negative (T, D^{-}), and triplets (T, D^{+}, D^{-}) are generated. This is achieved through the construction of a ground-truth matrix in the code implementation.

Each row of the ground-truth matrix represents the identifier of a protein, and each column represents the identifier of a drug. The cells in the ground-truth matrix are set to 1 for known interacting pairs and 0 for non-interacting or unknown pairs. This approach effectively utilizes the supervision of valuable labeled data and is suitable for real-world scenarios where test data may not have been encountered during training, thereby enriching the supervision information. These pairs are then mapped to latent space embeddings.

Given the protein target, drug compound latent features, and ground-truth matrix in a batch, we treat each target as an anchor and retrieve the marks of all drug compounds with respect to the anchor in the ground-truth matrix. We divide all compound types in the batch into positive and negative groups based on the marks, and consider all possible combinations between the two groups along with the anchor as the derived triplets for that target. This process is performed for each target, resulting in triplets for a batch.

The margin $m(t)$ defined in Eq. (12) of the main text plays a crucial role in training dynamics. Initially, $m(t)$ is set to a small value to ensure that the distance between the anchor and positive examples is significantly smaller than the distance between the anchor and negative examples, thereby avoiding penalties. This helps align protein features with corresponding drug features that are likely to interact during the early stages of training. As training progresses, a larger $m(t)$ relaxes the constraint between the anchor and positive examples and emphasizes maximizing the distance between the anchor and negative examples. This facilitates effective utilization of the supervision information in the later stages of training, further enhancing DrugLAMP's discriminative power in real-world scenarios.

This comprehensive implementation strategy ensures effective learning of drug-protein interactions while maintaining robust representation learning for each modality. The dynamic nature of the margin and the carefully designed loss functions work together to

achieve both good alignment of related pairs and clear separation of unrelated ones.

Additional Experiments

Visualization before and after 2C2P

To better illustrate the role of the 2C2P module, we conducted t-SNE and UMAP visualizations in real-world scenario on the Human dataset, depicting the reduced final feature fed into the multi-layer perception. As shown in Fig. S2, subfigure a represents the visualization of the test set without the inclusion of the 2C2P module, while subfigure b displays the image with the addition of 2C2P. Each point represents a drug-target pair, with colors indicating the label of interaction. We observed that the incorporation of the 2C2P module separates points between different labels and aggregates points with the same label. This visually demonstrates the effectiveness of the 2C2P module.

Raw data sources for datasets in standard scenario

We constructed Fig. 2 within the constraints of the page width using the selected metrics. To further elucidate this matter, we include a supplementary table (Tab. S5) that presents raw data sources for each of the three datasets.

Ablation studies on multiple datasets

To verify the consistency of our model's component contributions across different datasets, we performed ablation studies on both the BioSNAP and Human datasets in real-world scenarios. Tab. S6 shows the results for both datasets.

These results demonstrate that the contributions of each component in DrugLAMP are consistent across different datasets, reinforcing the robustness and generalizability of our model architecture. The similar patterns observed in both the BioSNAP and Human datasets suggest that the design choices in DrugLAMP are effective across varying data distributions and characteristics.

References

Cao, D. *et al.* (2013) propy: a tool to generate various modes of Chou ’ s PseAAC. Bioinformatics, 29(7), 960-962.

Chen, L. *et al.* (2020) TransformerCPI: improving compound – protein interaction prediction by sequence-based deep learning with self-attention mechanism and label reversal experiments. Bioinformatics, 36(16), 4406-4414.

Bai, P. *et al.* (2023) Interpretable bilinear attention network with domain adaptation improves drug – target prediction. Nature Machine Intelligence, 5(2), 126-136.

Bai, P. *et al.* (2021) Hierarchical clustering split for low-bias evaluation of drug-target interaction prediction. In 2021 IEEE International Conference on Bioinformatics and Biomedicine (BIBM) (pp. 641-644). IEEE.

Rogers, D. and Hahn, M. (2010) Extended-connectivity fingerprints. Journal of chemical information and modeling, 50(5), 742-754.

Figures

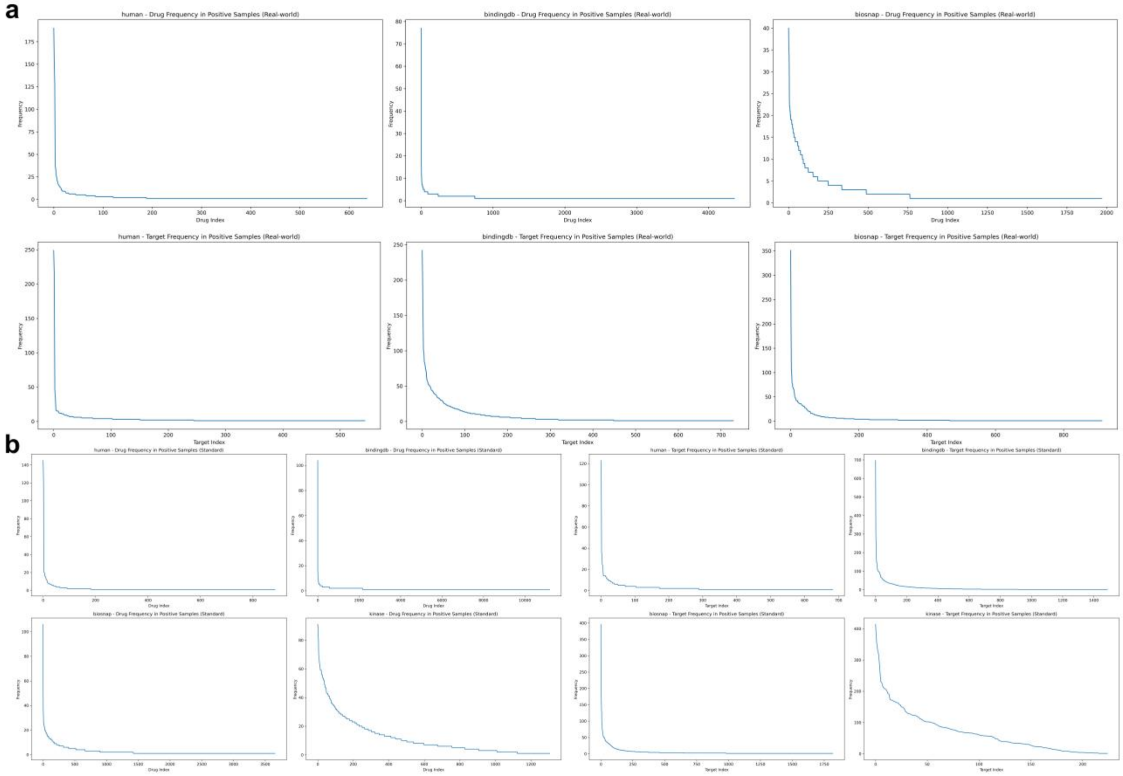

Figure S1. Frequency of drug and target occurrences in positive samples across different scenarios. a, Frequency of drug and target occurrences in positive samples under three real-world scenarios. **b,** Frequency of drug and target occurrences in positive samples under standard scenarios. Kinase exhibits the least bias compared to the real-world scenarios.

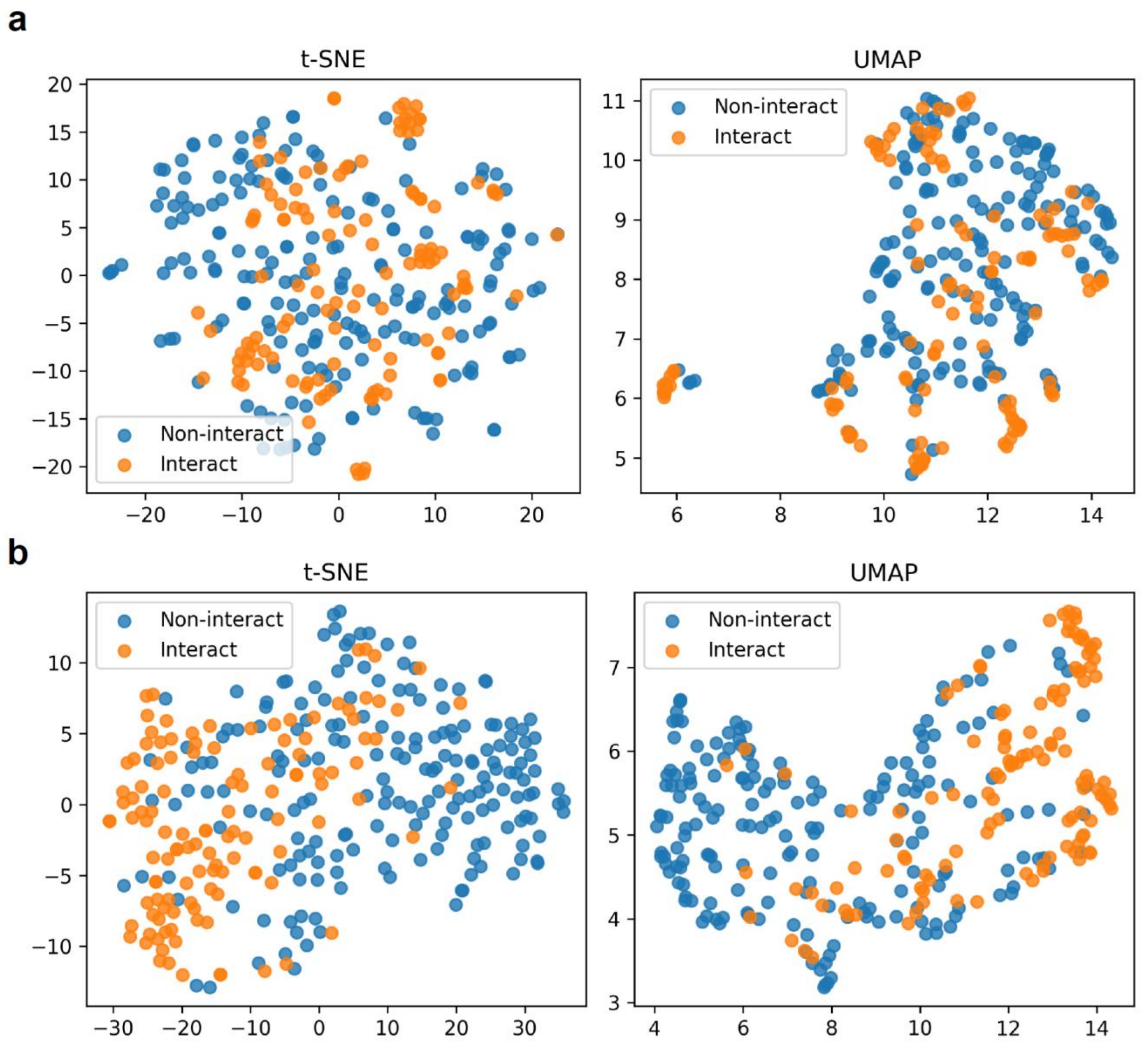

Figure S2. t-SNE and UMAP visualizations illustrating the role of the 2C2P module. **a,** Visualization of the test set features without the 2C2P module. Each point represents a drug-target pair, with colors indicating the label of interaction. **b,** Visualization of the test set features with the 2C2P module incorporated.

Algorithm 1 2C2P

Input: $E^D, M^D, S^P_{repeat}, M^P, ProtFeatExtractor, \text{Ada}, ff_D, ff_P$

function $\textsc{BatchUpdating}(E^D, M^D, S^P_{repeat}, M^P)$
  # Calculate the intra- and inter-modality losses.
  $L_{DSS}, L_{PSS} \leftarrow \textsc{DrugSSLoss}(E^D, M^D),\ \textsc{ProteinSSLoss}(S^P_{repeat}, M^P)$
  # Get the features of the current batch for cross-modality loss.
  $E^P_{pocket} \leftarrow \text{Pock}(ProtFeatExtractor(S^P_{repeat}))$
  $M^D_{ff}, M^P_{ff} \leftarrow ff_D(M^D), ff_P(\text{Ada}(\text{Pock}(M^P)))$
  # Calculate the cross-modality and 2C2P losses.
  $L_{CM} \leftarrow \textsc{TripletMargin-DistanceLoss}(E^D, M^D_{ff}, E^P_{pocket}, M^P_{ff})$
  $L_{2C2P} \leftarrow (1-\alpha)L_{CM} + \alpha(L_{DSS} + L_{PSS})$
  # Update the network.
  $\textsc{Backward-Update}(ProtFeatExtractor, \text{Ada}, ff_D, ff_P, L_{2C2P})$
end function

function $\textsc{DrugSSLoss}(E^D, M^D)$
  # Get the projected and predicted latents.
  $E^D_{Proj}, M^D_{Proj} \leftarrow ProjNet(E^D), ProjNet_{PLM}(M^D)$
  $E^D_{Pred}, M^D_{Pred} \leftarrow Predictor(E^D_{Proj}), Predictor(M^D_{Proj})$
  $E^{D^*}_{Proj}, M^{D^*}_{Proj} \leftarrow E^D_{Proj}.detach(), M^D_{Proj}.detach()$
  # Calculate the drug similarity loss.
  $L_{DSS} \leftarrow DS(E^D_{Pred}, M^{D^*}_{Proj})/2 + DS(E^{D^*}_{Proj}, M^D_{Pred})/2$
  return L_{DSS}
end function

function $\textsc{ProteinSSLoss}(S^P_{repeat}, M^P)$
  # Get the masking ground-truth with probability.
  $S^{P^*}_{repeat}, labels \leftarrow \textsc{MaskWithProb}(S^P_{repeat}, probability)$
  $E^{P^*}_{repeat} \leftarrow ProtFeatExtractor(S^{P^*}_{repeat})$
  # Get the logits and calculate the cross-entropy loss.
  $logits_S, logits_M \leftarrow Lin(S^{P^*}_{repeat}), Lin_{PLM}(M^P)$
  $L_{PSS} \leftarrow CE(logits_S, labels)/2 + CE(logits_M, labels)/2$
  return L_{PSS}
end function

function $\textsc{TripletMargin-DistanceLoss}(E^D, M^D_{ff}, E^P_{pocket}, M^P_{ff})$
  # Construct ground-truth matrix.
  $mat_{GT} \leftarrow \textsc{ConstructByBatchMeta}(metadata)$
  # Compute latent representations.
  $lats_D \leftarrow Lin^D(Embed^D(E^D) \oplus Embed^D_{PLM}(M^D_{ff}))$
  $lats_P \leftarrow Lin^P(Embed^P(E^P_{pocket}) \oplus Embed^P_{PLM}(M^P_{ff}))$
  # Derive triplets and calculate the triplet margin-distance loss.
  $a, p, n \leftarrow \textsc{DeriveTripletsForBatch}(lats_D, lats_P, mat_{GT})$
  $L_{CM} \leftarrow \frac{1}{N_{tri}} \sum_{i=1}^{N_{tri}} \max(\text{dist}(a_i, p_i) - \text{dist}(a_i, n_i) + m, 0)$
  return L_{CM}
end function

Algorithm 1.

Tables

Table S1. Common notation table

Notations	Description
S^D, S^P	sequence of drug, protein
S^D_{trim}, S^P_{repeat}	pre-processed sequence of drug, protein
$E^D(input\ to\ PGCA)$	drug feature after extraction
E^P	protein feature after extraction by S^P_{repeat}
$E^P_{pocket}(input\ to\ PGCA)$	protein pocket feature by E^P
M^D	drug feature after PLM by S^D_{trim}
$M^D_{ff}(input\ to\ PGCA)$	drug feature after feed forward layers
M^P	protein feature after PLM by S^P_{repeat}
M^P_{pocket}	protein pocket feature by M^P
M^P_{ada}	protein feature after adaptor by M^P_{pocket}
$M^P_{ff}(input\ to\ PGCA)$	protein feature after feed forward layers
F^E_{mixed}, F^M_{mixed}	mixed features of extractor and PLM after PGCA
F_{final}	final feature after PMMA

Abbreviation	Full name
DTI	Drug-Target Interaction
PLM	Pretrained Language Model
DrugLAMP	P**L**M-**A**ssisted **M**ulti-modal **P**rediction
PGCA	Pocket-guided Co-attention
PMMA	Paired Multi-modal Attention
2C2P	Contrastive Compound-Protein Pre-training
GCN	Graph Convolutional Network
SVM	Support Vector Machine
RF	Random Forest
AUROC	Area Under the Receiver Operating Characteristic Curve
AUPRC	Area Under the Precision-Recall Curve
ADE	Adverse Drug Event
SSL	Self-Supervised Learning
MLM	Masked Language Modeling
MHA	Multi-Head Attention
MLP	Multi-Layer Perceptron

Table S2. Experimental dataset statistics

Dataset	#Drug	#Protein	#Interaction	
			Positive	Negative
Human	2726	2001	3364	3364
BioSNAP	4505	2181	13830	13634
BindingDB	14643	2623	20674	28525
Kinase	1644	229	23190	88047

Table S3. The split details of experimental datasets

Dataset	Scenario	#Train		#Valid		#Test	
		Positive	Negative	Positive	Negative	Positive	Negative
Human	Standard	1846	2351	251	349	536	664
	Cold-start	1815	1638	59	96	121	190
BioSNAP	Standard	9684	9540	1398	1349	2748	2745
	Cluster-start	5001	4765	455	452	455	452
BindingDB	Cluster-start	5640	9288	826	953	826	953
Kinase	Standard	15289	57952	3911	14400	3990	15695

Table S4. Size of the ten largest clusters in cluster-start split datasets

Dataset	Object	# 1	# 2	# 3	# 4	# 5	# 6	# 7	# 8	# 9	# 10
BindingDB	Drug	598	460	304	290	253	250	203	202	198	158
BioSNAP	Drug	294	267	75	68	36	35	28	26	24	24
BindingDB	Protein	17	15	15	12	10	10	10	9	9	8
BioSNAP	Protein	8	8	8	6	5	4	4	4	4	4

Table S5. Common performance comparison on the three datasets in standard scenarios (statistics over five random runs)

Metrics	BioSNAP				
	AUROC	AUPRC	Accuracy	Sensitivity	Specificity
SVM	0.862	0.864	0.777	0.711	0.841
RF	0.860	0.886	0.804	0.823	0.786
DeepConv-DTI	0.886	0.890	0.805	0.760	0.851
GraphDTA	0.887	0.890	0.800	0.745	0.854
MolTrans	0.895	0.897	0.825	0.818	0.831
Kang et al.	0.910	0.900	-	**0.862**	0.847
DrugBAN	0.903	0.902	0.834	0.820	0.847
DLM-DTI	0.914	0.914	-	0.848	0.844
DrugLAMP	**0.917**	**0.922**	**0.850**	0.844	**0.855**

Metrics	Human	
	AUROC	AUPRC
SVM	0.940	0.920
RF	0.952	0.953
DeepConv-DTI	0.980	0.981
GraphDTA	0.981	0.982
MolTrans	0.980	0.978
DrugBAN	0.982	0.980
DrugLAMP	**0.985**	**0.983**

Metrics	Kinase	
	AUROC	AUPRC
GraphDTA	0.434	0.171
TransformerCPI	0.598	0.280
DrugLAMP	**0.631**	**0.341**

Table S6. Module ablation studies on BioSNAP and Human datasets in real-world scenarios (statistics over five random runs)

Variants	$\text{Human}_{\text{Cold}}$		$\text{BioSNAP}_{\text{Cluster}}$	
	AUROC	AUPRC	AUROC	AUPRC
$\text{DrugLAMP}_{\text{CM w/o PLM}}$	0.829	0.761	0.589	0.591
$\text{DrugLAMP}_{\text{CM w/o FE}}$	0.835	0.765	0.624	0.593
$\text{DrugLAMP}_{\text{CM w/o repeat}}$	0.841	0.749	0.736	0.761
$\text{DrugLAMP}_{\text{CM w/o PGCA}}$	0.837	0.767	0.639	0.621
$\text{DrugLAMP}_{\text{CM w/o PMMA}}$	0.846	0.781	0.683	0.713
$\text{DrugLAMP}_{\text{CM w/o 2C2P}}$	0.857	0.795	0.739	0.760
$\text{DrugLAMP}_{\text{CM}}$	**0.860**	**0.814**	**0.739**	**0.768**
